# Homologous recombination, cancer and the ‘RAD51 paradox’

**DOI:** 10.1093/narcan/zcab016

**Published:** 2021-05-17

**Authors:** Gabriel Matos-Rodrigues, Josée Guirouilh-Barbat, Emmanuelle Martini, Bernard S Lopez

**Affiliations:** Université de Paris, INSERM U1016, UMR 8104 CNRS, Institut Cochin, Equipe Labellisée Ligue Contre le Cancer, F-75014, France; Université de Paris, INSERM U1016, UMR 8104 CNRS, Institut Cochin, Equipe Labellisée Ligue Contre le Cancer, F-75014, France; Université de Paris and Université Paris-Saclay, Laboratory of Development of the Gonads, IRCM/IBFJ CEA, UMR Genetic Stability, Stem Cells and Radiation, F-92265 Fontenay aux Roses, France; Université de Paris, INSERM U1016, UMR 8104 CNRS, Institut Cochin, Equipe Labellisée Ligue Contre le Cancer, F-75014, France

## Abstract

Genetic instability is a hallmark of cancer cells. Homologous recombination (HR) plays key roles in genome stability and variability due to its roles in DNA double-strand break and interstrand crosslink repair, and in the protection and resumption of arrested replication forks. HR deficiency leads to genetic instability, and, as expected, many HR genes are downregulated in cancer cells. The link between HR deficiency and cancer predisposition is exemplified by familial breast and ovarian cancers and by some subgroups of Fanconi anaemia syndromes. Surprisingly, although RAD51 plays a pivotal role in HR, i.e., homology search and in strand exchange with a homologous DNA partner, almost no inactivating mutations of *RAD51* have been associated with cancer predisposition; on the contrary, overexpression of RAD51 is associated with a poor prognosis in different types of tumours. Taken together, these data highlight the fact that RAD51 differs from its HR partners with regard to cancer susceptibility and expose what we call the ‘RAD51 paradox’. Here, we catalogue the dysregulations of HR genes in human pathologies, including cancer and Fanconi anaemia or congenital mirror movement syndromes, and we discuss the RAD51 paradox.

## Graphical Abstract

**Graphical abstract:**
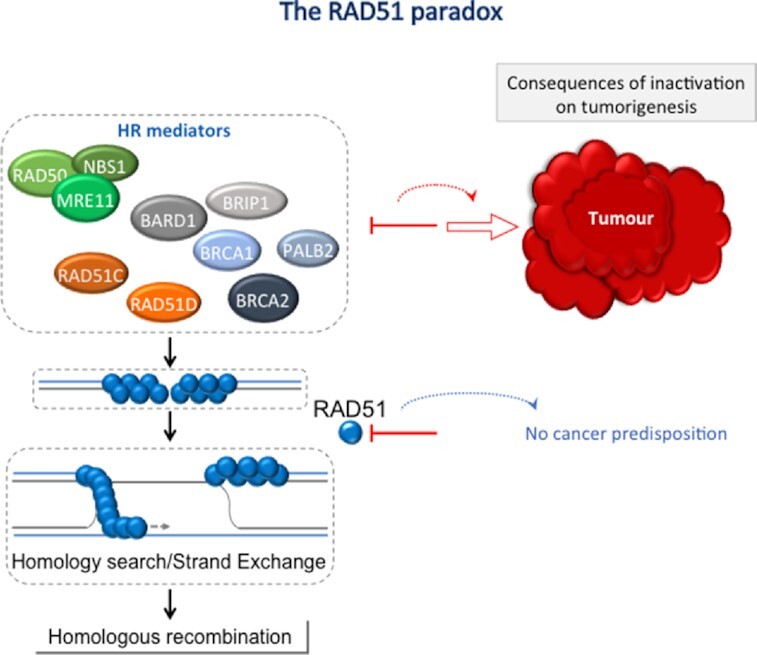
The RAD51 paradox.

## INTRODUCTION

Genomes are routinely challenged by exogenous and endogenous stresses, leading to genetic instability that can fuel oncogenesis (1,2). To preserve genome integrity, cells have developed the DNA damage response (DDR) that coordinates cell cycle progression and DNA repair.

Homologous recombination (HR), a process that is highly conserved throughout evolution, plays a prime role in genome stability/diversity. HR is involved in the repair of DNA double-strand breaks (DSBs) and DNA interstrand crosslinks (ICLs) and in the protection and resumption of arrested replication forks (3). In particular, HR suppression alters replication dynamics (4–6). Notably, the activation of the DDR has been observed during the pre/early stages of cancer as a result of endogenous replication stress (7,8), suggesting the potential role of HR as a replication escort in preventing cancer initiation. Moreover, DSBs and ICLs are also important sources of genetic instability, and thus the role of HR in their repair also favours the maintenance of genome stability. Therefore, HR is widely considered a tumour suppressor pathway.

**Figure 1.**
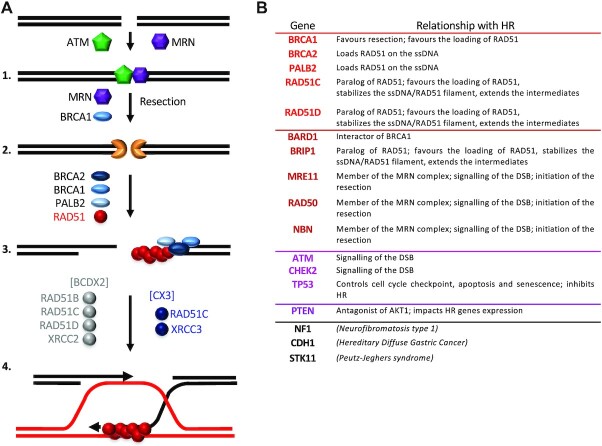
Germline mutations of HR genes in familial breast/ovarian cancers. (**A**) The HR molecular steps and HR factors affected in cancer. DNA double-strand break repair by HR can be summarized as follows: (1) Double-strand break (DSB) recognition and signalling by the MRN (MRE11-RAD50-NBS1 complex) and ATM. (2) MRN and BRCA1 favour the initiation of the DNA ends generating 3′ single-stranded DNA (ssDNA) overhangs, which are covered and protected by RPA (not represented). (3) Then, BRCA1/BRCA2/PALB2 displace RPA and replace it with RAD51, forming the ssDNA/RAD51 filament. (4) Homology search and strand exchange. The ssDNA/RAD51 filament promotes the homology search and the invasion of a homologous sequence (red line), thus representing the commitment step of the HR pathway; the ssDNA/RAD51 filament is thus the active species of HR. The RAD51 paralogs, which are associated in two distinct complexes RAD51B-RAD51C-RAD51D-XRCC2 [BCDX2] and RAD51C-XRCC3 [CX3], favour the assembly and the stabilization of the ssDNA/RAD51 filament and of the HR intermediates; they also can participate in the steps downstream of the homology search. The last step of HR (not represented) is the resolution of the HR intermediate created by the action of the ssDNA/RAD51 filament, resulting in gene conversion associated or not with crossing over. (**B**) List of the genes mutated in familial breast or ovarian cancer. Red: core HR genes directly involved in the loading of RAD51 on ssDNA and the stabilization of the ssDNA/RAD51 filament. Deep red: accessory HR genes; purple: DDR genes that can impact HR; deep purple: other genes that can affect HR.

In support of this notion, several HR genes are mutated in tumours. Paradoxically, despite extensive studies, the inactivation of RAD51, which performs the sequence homology search (i.e. the central step of HR that gives the process its name, see Figure 1A) has not been found to be related to cancer development. Notably, *RAD51* inactivating mutations are absent in familial breast and ovarian cancer (Figure 1B). In contrast, overactivation or overexpression of RAD51 has been described in different types of cancer. Moreover, germline mutations in several HR genes are responsible for subgroups of Fanconi anaemia (FA), a rare autosomal recessive syndrome leading to developmental defects and malignancies (9). However, to date, there has been no case of a patient with FA-R (*RAD51* mutation) developing cancer. Hence, compared to all its HR partners, RAD51 is an enigma, especially considering its central role in HR (see Figure 1A). Moreover, the fact that *RAD51* is an essential gene in mammals has hindered *in vivo* analysis of RAD51 functions.

Here, we discuss the relationships between HR genes and human pathologies, including cancers. Then, we discuss the ‘RAD51 paradox’.

## MUTATION AND DOWNREGULATION OF HR GENES IN HUMAN PATHOLOGIES: THE RAD51 PARADOX

HR is downregulated in different cancer contexts, including germline mutations in inherited breast or ovarian cancer (Figure 1) or in FA subgroups. Some *RAD51* mutations have been detected in different types of sporadic cancer, but whether they cause tumourigenesis remains unclear (10). Finally, *RAD51* mutations have been identified in congenital mirror movement syndrome but were not associated with cancer predisposition (11,12).

### Familial breast and ovarian cancer

Heterozygous germline mutations in different genes confer predisposition to breast or ovarian cancers (Figure 1B) (13). Genes involved in DDR appear to be markedly overrepresented; specifically, the main represented pathway is HR (Figure 1B). Indeed, several genes that directly control HR are mutated (*BRCA1, BRCA2, PALB2, RAD51C, RAD51D, BARD1, BRIP1, MRE11, RAD51, NBN)*, as are other genes that indirectly impact HR, such as the DDR-controlling genes ATM and CHEK2. The association between mutations of the HR mediators *XRCC2* and *XRCC3* and cancer predisposition remains controversial. Moreover, TP53 precludes HR independently of its roles in the cell cycle and apoptosis (for review, see (14)). PTEN has also been proposed to compromise HR (15,16), but this remains contentious (17,18). As a consequence, the HR genes *BRCA1, BRCA2, PALB2, RAD51C, RAD51D, BARD1, BRIP1, MRE11, RAD51* and *NBN* are included in several hereditary breast/ovarian cancer screening panels to evaluate the tumour for HR deficiency and predict its response to chemotherapy (19).

However, in spite of the importance of HR alteration in hereditary breast/ovarian cancer, mutations of the pivotal HR player RAD51 are surprisingly absent from the lists of genes predisposing individuals to breast or ovarian cancer (Figure 1A and B).

While many cancer-related mutations affect HR genes, only two germline mutations in *RAD51* have been identified, and their impact on cancer risk remains to be established. The RAD51 E258A mutation is a dominant negative germline variant that was identified in breast carcinoma and maps to the interface region between the N-terminal and RecA homology domains of RAD51 (20). The RAD51 R150Q mutation leads to reduced ssDNA and dsDNA binding abilities (21). However, the mutation is not clearly associated with cancer incidence (22). Therefore, unlike its direct HR partners, RAD51 has not been classified as causal for cancer. Consequently, *RAD51* is not included in genetic diagnostic tests or in screening for tumour chemotherapy response.

### Fanconi anaemia

FA is a rare autosomal recessive syndrome that is associated with developmental defects and malignancies (9). Homozygous or heteroallelic germline mutations in several HR genes have been described in FA subgroups, namely, *BRCA1* (FA-S), *BRCA2* (FA-D1), *PALB2* (FA-N), *RAD51C* (FA-O), *XRCC2* (FA-U) and *RAD51* (FA-R). Remarkably, FA patients with *RAD51* mutation (FA-R) do not develop cancers, although they present other developmental anomalies related to FA (23–25). However, only a few FA-R patients have been reported to date.

Nevertheless, both FA syndrome and familial breast and ovarian cancer reveal the association of HR gene germline mutations with cancer susceptibility, with the exception of the central HR player RAD51.

### Sporadic cancers

#### Mutations of HR genes in sporadic cancers

In addition to being implicated in hereditary cancers, mutations in HR genes were also observed in a large panel of sporadic cancers. Two recent studies in 64 791 and 113 927 women respectively confirmed that mutations in HR genes BRCA1, BRCA2, PALB2, BARD1, RAD51C and RAD51D correlate with increased breast cancer incidence (26,27). More generally, the sequencing of a panel of 52 426 tumours, including melanoma, hepatocellular carcinoma, and endometrial, gastroesophageal, ovarian, colorectal, biliary tract, bladder, breast and pancreatic cancers, showed that 15–34% of tumours exhibited mutations in HR or DDR genes (10). Interestingly, in this panel, only the *BRCA1, BRCA2, PALB2* and *BRIP1* genes were found to be mutated, and no mutation was observed in *BARD1, RAD51C* or *RAD51*. The *BRCA1* and *BRCA2* genes are mutated in 5–6% of breast cancers and 16% and 6% of ovarian cancers, respectively. Mutations in not only *BRCA1/2* but also *RAD51C, RAD51D, PALB2, BARD1* and *BRIP1* are now also screened in clinics to identify HR deficiency, which has recently become a key criterion for treatment with PARP inhibitors. It is noteworthy that no screens include *RAD51*, as it does not appear to be a frequently mutated gene in these pathologies.

In some isolated cases, mutations of *RAD51* have been found in tumours. Most of these mutations were classified as variants of unknown significance, but some were functionally characterized (F86L, D149N, Q268P, Q272L) and determined to affect one of the functions of RAD51 (ATPase activity, DNA binding, strand exchange activity and/or thermal stability) (28). However, the HR process itself was not evaluated in cells (for review see (29)). All of the mutations were found in breast cancers, with the exceptions of Q268P (lung cancer) and Q272L (kidney cancer). An additional variant, RAD51 G151D, was found in a triple-negative breast cancer (30,31), but remarkably, this mutation leads to overactivation of RAD51 activity instead of inactivation (30). Finally, it is not known whether these few RAD51 mutations are actually causal for cancer.

#### Expression of HR genes in sporadic cancers

Expression of HR factors may often be downregulated by promoter methylation rather than (or in addition to) mutations; for instance, the BRCA1 and BRCA2 promoters are methylated in 20% and 5% of epithelial ovarian carcinomas, respectively (32).

In contrast, overexpression of RAD51 is common in many cancers, including cervical, non-small cell lung, breast, ovarian and pancreatic cancers, melanoma and glioblastoma. This overexpression is associated with poor prognosis as a consequence of increased ability to repair lesions induced by DNA-damaging therapeutic agents (33–38); cancer cells overexpressing RAD51 could be selected during tumour progression because of this survival advantage. RAD51 overexpression of is generally due to overactivation of the promoter in cancer cells (38). In contrast with the general pattern of HR-inactivating mutations promoting breast cancer, RAD51 overexpression is associated with poor prognosis (https://www.proteinatlas.org). Therefore, RAD51 is a pharmacological target, and RAD51 inhibitors are being developed (29).

#### Regulators of HR are dysregulated in cancer

AKT1 is an oncogenic kinase that is activated in numerous types of cancers. AKT1 exercises its oncogenic activity through the stimulation of proliferation associated with the inhibition of apoptosis. Remarkably, AKT1, which is negatively regulated by PTEN (one of the genes mutated in hereditary breast and ovarian cancer), also inhibits HR through the cytoplasmic sequestration of BRCA1 and RAD51, resulting in at least a BRCA1 defective-like phenotype (39). The fact that AKT1 is activated in 40–60% of sporadic breast or ovarian cancers establishes a potential metabolic link between familial and sporadic breast and ovarian cancer (40).

BCL-2 is one of the most important antiapoptotic genes, and although it facilitates tumour cell survival and proliferation, overexpression of BCL-2 has been consistently associated with good prognosis (50). BCL-2 downregulates HR through the mislocalization of BRCA1 to the mitochondrial membrane (41,42). It may be proposed that the enhanced survival resulting from apoptosis inhibition is at least in part compensated by a lower capacity to resist treatment.

A translocation between chromosomes 9 and 22 (also known as the Philadelphia chromosome) resulting in expression of the BCR-ABL tyrosine kinase is found in chronic myelogenous leukaemia (CML). BCR-ABL expression leads to overexpression of RAD51 and several RAD51 paralogues via STAT5-dependent transcription and inhibition of caspase-3-dependent cleavage, thus resulting in a hyper-RAD51-like phenotype (43).

TP53 is the most frequently mutated gene in cancers. Since TP53 precludes HR, particularly targeting RAD51, mutation of TP53 also leads to elevated HR levels (for review, see (14)).

### Congenital mirror movement syndrome

Congenital mirror movement (CMM) disorder is a rare disorder that impairs the patient's capacity to perform normal daily tasks that require bimanual coordination (11,12). Multiple CMM patients (*n* = 32) have been reported to have *RAD51* mutations, usually RAD51 haploinsufficiency. These patients did not show additional developmental malformations, and no mutation in other HR factors has been found in CMM. In an investigation of the function of RAD51 in the context of CMM, a study using primary mouse cortical neurons suggested that RAD51 can negatively regulate neuronal axon growth (44). Importantly, CMM patients bearing RAD51 mutations (but no mutations of other HR genes) do not exhibit cancer predisposition.

The authors propose a cytoplasmic function for RAD51 in neuronal guidance, but the details of this mechanism remain to be characterized. Nevertheless, these data suggest an additional specific function of RAD51 that might contribute to the RAD51 paradox.

## THE RAD51 PARADOX IN CANCER: HYPOTHESES AND SPECULATIONS

**Figure 2.**
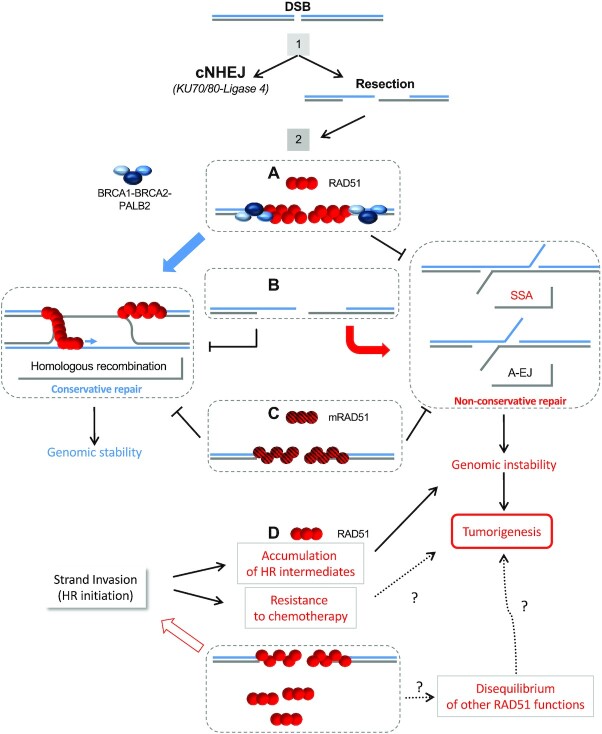
Roles of RAD51 in DSB repair pathway selection and consequences for genomic instability. The choice of the DSB repair pathway occurs in two steps (52, 53): 1, competition between canonical non-homologous end-joining (cNHEJ), which is conservative (53), and resection leading to a 3′ ssDNA; 2, competition between conservative HR and non-conservative single-strand annealing (SSA) or alternative end-joining (A-EJ) on the 3′ ssDNA. Resection can reveal complementary single-stranded DNA; their annealing results in SSA (long sequences) or A-EJ (microhomology-mediated) with loss of the intervening sequence. Thus, SSA and A-EJ are non-conservative, leading to genomic instability. (**A**) Loading of wild-type RAD51 (red circles) on ssDNA triggers conservative HR through its enzymatic search for homology and strand exchange activity and thus maintains genome stability. In addition, the presence of RAD51 on ssDNA prevents the annealing of the complementary ssDNA, protecting genome stability against non-conservative repair. (**B**) The absence of RAD51 on ssDNA does not allow HR and makes DNA accessible to alternative and non-conservative SSA and A-EJ, fostering genomic instability. (**C**) The loading of mutant mRAD51 (hatched red circles) proteins with maintained DNA binding capacities, yet unable to perform strand exchange, will result in defective HR without stimulation of alternative non-conservative repair. Non-conservative repair generates genomic instability that can fuel tumourigenesis. (**D**) Overexpression of RAD51 might initiate more strand exchange and HR events, resulting in resistance of DNA damage-based chemotherapy. In addition, this putative stimulation of strand exchange might result in the accumulation of unresolved HR intermediates that generate genetic instability. RAD51 overexpression might result in disequilibrium of other HR functions and cell homeostasis, leading to tumourigenesis.

Several mechanisms that can operate together or in parallel might account for the RAD51 paradox:

- *RAD51 functions that are independent of RAD51 mediators/loaders*. CMM syndrome suggests that RAD51 could have BRCA/PALB2-independent functions, potentially cytoplasmic. The alteration of these functions, in addition to defective genome maintenance, might be so toxic that cancer cell proliferation cannot occur. Moreover, RAD51 exhibits some BRCA-independent DNA processing activities. Indeed, in addition to its function during DSB repair, RAD51 has important functions during replication, which can be independent of its classical loading factors. Blocked replication forks can be regressed, generating a so-called ‘chicken foot’ structure, and RAD51 is thought to directly promote such structures in a BRCA2- and PALB2-independent manner (45–48). The protein complex MMS22L-TONSL is recruited to blocked replication forks, and its inactivation decreases survival after replication fork stall. This complex is involved during replication stress-mediated HR and gene conversion (49–51). MMS22L-TONSL binds directly to RAD51, and its inactivation decreases RAD51 recruitment to blocked replication forks (50). It was proposed that MMS22L-TONSL could load RAD51 onto the ssDNA in blocked replication forks, replacing RPA through a BRCA2/PALB2-independent mechanism (46,50). Based on this evidence, one could propose that because RAD51 possesses both BRCA1/2/PALB2-dependent and BRCA1/2/PALB2-independent functions during replication, its inactivation would be more toxic than that of one of the loading factors, thus impairing the proliferation of tumour cells.
- *Stimulation of alternative non-conservative DNA repair pathways*. The choice of the appropriate DSB repair pathway is important for genome stability maintenance. Different processes cooperate or compete to achieve DSB repair, and inactivation of HR can have several different outcomes and consequences depending on the process involved. The selection of the DSB repair mechanism occurs in two steps (Figure 2). First, competition between canonical non-homologous end-joining and resection that generates the 3′ ssDNA; second, competition between HR and single-strand annealing (SSA) or alternative end-joining (A-EJ) on the 3′ ssDNA (52,53). Importantly, HR is mainly conservative, while SSA and A-EJ are non-conservative and cause genome instability because they ineluctably involve the loss of the sequences surrounding the DSB. RAD51 performs its role(s) in genome stability maintenance at the second step through both enzymatic and non-enzymatic processes (Figure 2). The enzymatic strand exchange activity of the ssDNA/RAD51 filament triggers the search for homology and strand exchange with a homologous DNA partner. This ultimately leads to HR-mediated repair and resumption of arrested replication forks, which are conservative pathways that preserve genome stability. In parallel, RAD51 protects arrested replication forks and prevents non-conservative DSB repair processes through DNA occupancy (Figure 2), independent of strand exchange activity, in a non-enzymatic manner (54,55). Indeed, the loading of a RAD51 molecule that cannot perform strand exchange still protects against extensive degradation of arrested replication forks (54) as well as the annealing of complementary ssDNA (55), the central step of non-conservative SSA and A-EJ (see Figure 2). Consequently, the absence of RAD51 protein on damaged DNA may result in the stimulation of alternative non-conservative DNA repair pathways that increase genetic instability. Therefore, ablation of the mediators/loaders of RAD51, i.e., the partners of RAD51, results not only in the suppression of conservative HR but also in the concomitant stimulation of non-conservative SSA and A-EJ (55–58). In addition, the stimulation of alternative repair pathways allows partial rescue from the cell viability defects caused by the failure of HR, thus increasing the viability of cells with increased genetic instability. Therefore, it can be suggested that HR ablation alone would not be sufficient for tumour development and/or viability and that the association with the stimulation of the non-conservative pathways would be required for tumourigenesis. PolQ is overexpressed in cancers and is predictive of a poor prognosis (59). The fact that PolQ removes RAD51 from the filament, thus suppressing HR (60), supports the idea that HR suppression is tumourigenic. However, by removing RAD51 from the DNA, PolQ also stimulates A-EJ (60), overexpression of PolQ might be associated with both the inhibition of conservative HR and non-conservative pathway stimulation (A-EJ).

In this context, the consequences for cancer outcome might depend on the way HR is inhibited and the presence versus the absence of RAD51 protein on damaged DNA. According to this hypothesis, only mutations that suppress RAD51 expression or its loading and/or stabilization on damaged DNA (such as mutations in BRCA1, BRCA2 or PALB2 or other HR factors) should confer cancer predisposition. Note that another essential gene, KNL1 (Kinetochore scaffold 1), which is involved in mitotic spindle assembly and the associated checkpoint, is close to the *RAD51* gene on chromosome 15 (both genes are in cytogenetic band 15q15.1). Homozygous codeletion of these two genes is likely too deleterious for cell viability and especially unlikely to be able to support high levels of proliferation as in cancer cells. This would then decrease the frequency of possible *RAD51* deletions. Moreover, the hypothesis implies that *RAD51* missense mutations that abolish HR but retain the DNA-binding capacity of RAD51 should not promote cancer. These factors would decrease the probability and frequency of carcinogenic *RAD51* mutations, especially compared to those of its HR partners, which are not subject to such restrictions.

Finally, we cannot exclude the possibility that other reasons (e.g., different stability of mutant RAD51 proteins or mRNA, different sensitivity of tumours to detection by the immune system, and many other possibilities…) that are not yet identified or characterized might also participate in the RAD51 paradox.

*Overexpression of RAD51*. Although RAD51 is not (or is rarely) found to be inactivated in cancer, its overexpression has been described in a wide variety of cancers, leading to poor prognosis (33–38). One hypothesis is that RAD51 overexpression confers resistance to chemotherapeutic agents that target DNA, accounting for the selection of such cells and for the poor prognosis (33–38). This hypothesis implies that overexpression of RAD51 initiates strand exchange events that result in increased HR activity, and thus resistance to DNA damaging agents (Figure 2D). One can propose that, in addition, unresolved HR intermediates accumulate, which can favour genetic instability (61). Finally, overexpression of RAD51 might disequilibrate other RAD51 functions, including cytoplasmic roles. This could alter cell homeostasis, fostering tumourigenesis and/or tumour progression (Figure 2D).

## CONCLUSIONS AND PERSPECTIVES

HR plays a central role in genome stability maintenance. Its suppression generates genome instability and should thus confer cancer predisposition. However, analysis of human pathologies reveals that the causal connection is in fact complex. Indeed, although suppression of most HR factors does trigger carcinogenesis, one important gene, *RAD51*, escapes this pattern. This is particularly remarkable because RAD51 promotes the pivotal steps of HR, i.e., homology searching and strand exchange with a homologous DNA partner. These steps quite literally define the HR process. In contrast, RAD51 has been found to be overexpressed or overactivated in tumours, which might result from the selection of tumour cells that are resistant to treatment with genotoxic agents. This suggests that the amount of RAD51 protein is a limiting factor for HR.

Mouse models should represent useful tools to experimentally address these questions *in vivo*. Unfortunately, most of the genes involved in the central step of HR, including *RAD51*, are essential, and their homozygous deletion leads to embryonic lethality in mice (62,63). To overcome these problems, elaborate strategies for partial HR or tissue-specific deletion have been designed (62). These models have confirmed that defects in genes that participate in different steps of HR trigger tumourigenesis. Surprisingly, despite its critical role in HR, there are no such alternative mouse models available for *RAD51*. Addressing the function of RAD51 *in vivo* will likely be key to solving the RAD51 paradox.

Different factors can be combined to account for the RAD51 paradox (see above). Among these factors, the presence *versus* absence of RAD51 on damaged DNA could influence commitment of a cell to a cancerous pathway. Indeed, the HR genes affected in cancer promote the formation and/or stabilization of the ssDNA/RAD51 filament. Depletion of these HR genes results in inefficient formation or stabilization of the ssDNA/RAD51 filament, making DNA accessible to alternative non-conservative repair processes that lead to increased genetic instability associated with partial compensation of the decreased viability. This raises the provocative question of whether HR suppression actually promotes cancer *per se* or whether oncogenesis in fact results from the stimulation of non-conservative pathways (or a combination of both). Addressing this question will be important to resolving the RAD51 paradox. Studying tumourigenesis when HR is inhibited in the absence of stimulation of alternative non-conservative pathways, when the non-conservative pathways are stimulated without altering HR, or when both pathways are inhibited should provide important clues to understanding why RAD51 disruption is not commonly found in cancers. These concepts will also be important to the design of strategies targeting RAD51 activity in cancer therapy. While such strategies should efficiently sensitize RAD51-overexpressing tumours to radiotherapy and chemotherapy, their use in other types of tumours might be more problematic because of the potential stimulation of non-conservative DNA repair pathways. An ideal strategy would be to repress HR and, in parallel, inhibit or at least ensure not to stimulate the non-conservative DNA repair pathway, thus avoiding the rescue of cancer cell viability and increased genetic instability.

BRCA2-independent functions of RAD51 may also play important roles in the RAD51 paradox. The full identification and characterization of RAD51 functions and the consequences of their inactivation on cell viability and carcinogenesis should also be informative for solving the RAD51 paradox.
